# Driver Attention Assessment Using Physiological Measures from EEG, ECG, and EDA Signals †

**DOI:** 10.3390/s23042039

**Published:** 2023-02-11

**Authors:** Taraneh Aminosharieh Najafi, Antonio Affanni, Roberto Rinaldo, Pamela Zontone

**Affiliations:** Polytechnic Department of Engineering and Architecture, University of Udine, Via Delle Scienze 206, 33100 Udine, Italy

**Keywords:** driver attention, electrodermal activity, electrocardiogram, electroencephalogram, blink rate, driving simulator

## Abstract

In this paper, we consider the evaluation of the mental attention state of individuals driving in a simulated environment. We tested a pool of subjects while driving on a highway and trying to overcome various obstacles placed along the course in both manual and autonomous driving scenarios. Most systems described in the literature use cameras to evaluate features such as blink rate and gaze direction. In this study, we instead analyse the subjects’ Electrodermal activity (EDA) Skin Potential Response (SPR), their Electrocardiogram (ECG), and their Electroencephalogram (EEG). From these signals we extract a number of physiological measures, including eye blink rate and beta frequency band power from EEG, heart rate from ECG, and SPR features, then investigate their capability to assess the mental state and engagement level of the test subjects. In particular, and as confirmed by statistical tests, the signals reveal that in the manual scenario the subjects experienced a more challenged mental state and paid higher attention to driving tasks compared to the autonomous scenario. A different experiment in which subjects drove in three different setups, i.e., a manual driving scenario and two autonomous driving scenarios characterized by different vehicle settings, confirmed that manual driving is more mentally demanding than autonomous driving. Therefore, we can conclude that the proposed approach is an appropriate way to monitor driver attention.

## 1. Introduction

According to the European Union annual accident report [1], during 2019 and considering EU member countries, 22,700 people died in crashes on roads, and more than 1.2 million were injured.Vehicle crashes on EU roads are estimated to cost EUR 280 billion yearly [2]. Driver error is known to be the main contributor to all road crashes [3,4]. Among the major causes of human error are inappropriate lookout [5], high speed [6], and inattention [7]. Inappropriate lookout includes surveillance error and looked-but-failed-to-see errors, which are in turn related to inattention. Thus, driver inattention is an important contributor to road crashes. Essentially, driver inattention is defined as inadequate attention or failure to pay attention to crucial driving tasks [8]. This may happen in the form of restricted attention when the driver is intoxicated, fatigued, or drowsy, or in the form of diverted attention when the driver is distracted by stimuli derived from sources other than driving tasks, such as navigation systems, mobile phones, passengers, or external sources [7].

Driving is a complex and resource-demanding activity for the driver. Essentially, it is a sensorimotor task that involves: (1) perception of environmental stimuli through the visual [9], vestibular [10], and somatosensory [11] systems; (2) processing of signals and planning of responses by the brain; and (3) transmission of signals by the central nervous system (CNS) to muscles in order to execute physical responses [12]. Therefore, drivers have an active role in the closed loop of environment perception and vehicle control activity. Naturally, their attention is essential to keeping the loop running safely and preventing accidents. Numerous techniques have been proposed by researchers [13,14,15] and car manufacturing companies to detect drivers’ attention state using driver monitoring systems (DMSs) as a part of advanced driving assistance systems (ADAS) [16,17]. In general, a DMS is designed to automatically detect the attention level of the driver and in case of necessity to warn them with various methods such as audio warnings or vibrations in order to prevent dangerous situations and crashes. Commonly, most DMSs employ various kinds of camera-based solutions along with image processing techniques to estimate the driver’s attention/inattention level. Although these techniques are very popular because of the simplicity of their installation and use, they can be less efficient when the vehicle is in motion due to vibrations created by its movement, potentially resulting in noisy images. Furthermore, while driving the background color of the scene is constantly changing, as is the light direction. This can create a partial or complete shadow on the driver’s face, making it difficult for such systems to recognize the face and its attributes. Additionally, the efficiency of such systems may vary due to the time of day or to weather conditions such as rain or snow because of variations in light intensity. Moreover, because most camera-based methods make use of ocular indices, if the driver is wearing spectacles or sunglasses, eye detection is more difficult for the system, and as a result it may not work properly.

Another approach to assess driver attention is based on the use of physiological measurements. These indicators can be acquired through various methods, including electroencephalography (EEG), electrocardiography (ECG), and electrodermal activity (EDA) measurements. Although these can be affected by different types of artifacts, as discussed in Section 5.3, they are generally exempt from the aforementioned concerns about weather and environmental conditions. Furthermore, physiological measures are known to be accurate [18] and reliable [19], and their combination in a multi-modal sensor fusion architecture can increase the robustness of the various measurements [20]. In addition, because the measurements are acquired directly from the subject’s body, they have low latency and high temporal resolution [21]. Several studies have presented physiological measures as valid sources for human cognitive state evaluation [22]. For example, in [23] the authors confirmed a negative correlation between attention and blink frequency. From [24], we know that EDA has a long history of use as an indicator of attention and arousal. The research study presented in [25] reported a relation between visual attention accuracy and increase in EEG beta frequency band power in the occipital region of the brain. The authors of [26] showed a positive correlation between increased anxiety and EEG beta power, as well as a surge in heart rate, during attentional tasks.

This study is an extension of our previous conference paper [27]. In that paper, we briefly described an experiment conducted at our university, hereinafter referred to as the first experiment, in which we acquired EEG signals from subjects while driving in a professional driving simulator and using our specially designed EEG headset. We then computed the blink rate by processing the EEG signals recorded from the frontal regions of the subject’s head. We noticed that the blink rate decreased during manual driving tasks with respect to autonomous driving; thus, we concluded that drivers had higher attention during manual driving as compared to autonomous driving. In this work, we present a new experiment, from now on referred to as the second experiment, in which we recorded the Skin Potential Response (SPR), ECG, and EEG signals from a different set of individuals in manual and autononomous driving scenarios. The SPR and ECG signal features have been shown to be good indicators of a subject’s mental state. As examples, in [28,29] these two physiological signals were employed to analyze the effect of traffic situations on subjects while driving in an urban area. In [30,31], we previously evaluated the emotional state of individuals wearing both SPR and ECG sensors driving along the same route and using a variety of car handling settings. Here, we report the results obtained when using SPR, ECG, and EEG signals together through a multisensor recoding system. In addition to the blink rate measures calculated using EEG frontal channels, in this novel experiment we evaluate the EEG beta band power, ECG heart rate, and EDA SPR measures in order to asses the attention level of drivers. We show that different driving scenarios induce different responses in the test subjects; in accordance with [27], the manual driving scenario impacts the subjects more, evidencing a more challenged mental state than in the autonomous scenario.

In summary, the main contributions of this paper are as follows. (1) We develop a system for assessing driver attention based on the analysis of various physiological signals, in particular, EEG, ECG, and EDA-SPR. The system consists of wireless wearable sensors, with their own firmware, and signal processing software. (2) Unlike other solutions proposed in the literature for assessing driver attention levels, which mostly rely on visual information, here we estimate the blink rate and the beta power on the basis of EEG, the heart rate on the basis of ECG, and SPR RMS measures on the basis of EDA. (3) We report the results of two experiments comparing autonomous and manual driving scenarios. These experiments demonstrate that drivers are more engaged during manual driving, and confirm the applicability of the proposed system in practical driving scenarios.

## 2. Materials and Methods

### 2.1. Sensor Description

**Figure 1:**
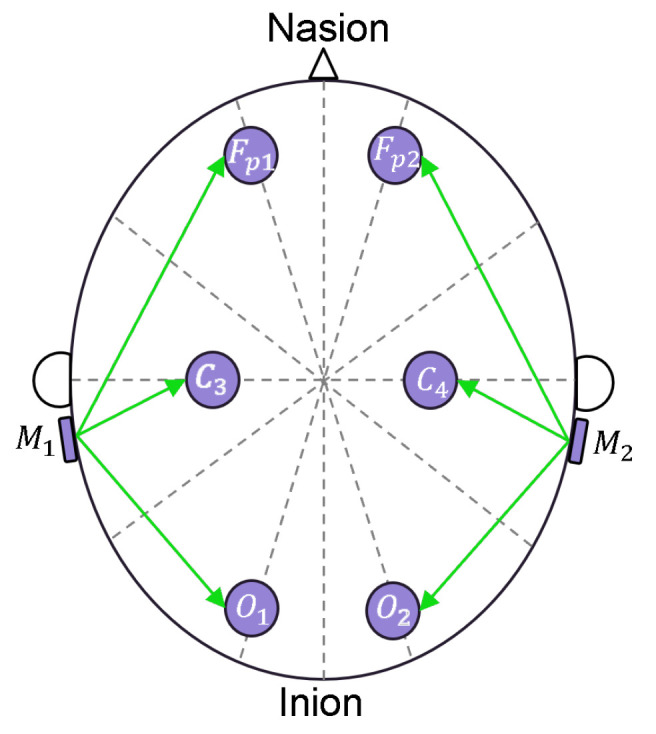
Placement of the EEG electrodes according to the 10/20 standard.

In this section, we describe the architecture of the sensors used in the experiments introduced above. In the first experiment, we used our own EEG headband design, which is characterized in [32]. To briefly describe its characteristics and specifications, the headband has six electrodes which acquire EEG signals from the scalp; two of them are located in the frontal region, two in the central region, and two in the occipital region. Referring to the 10–20 international system [33], the electrode positions are at the locations named Fp1, Fp2, C3, C4, O1, and O2. In Figure 1, the electrode locations are indicated with gray circles. The reference electrodes are shown placed at the locations of mastoids $M_{1}$ and $M_{2}$.

The circuit of the EEG sensor is composed of an analog section and a digital section. In the analog section, the signals are conditioned by means of six differential amplifiers that convert the differential voltages between each electrode and the reference electrodes $M_{1}$ and $M_{2}$ into high-level single-ended voltages. The low-level voltage of each channel is in the ±350 μV range, while the high-level output voltage spans up to 3.3 $V_{pp}$. Each differential amplifier is characterized according to these specifications in [32], showing a gain of 4210±35. The nonlinearity of the amplifiers results in their being on the order of 6 μV. The amplifiers are designed for band-pass behavior in the [0.8, 44] Hz range, with slopes of +40 dB/dec and −60 dB/dec for the lower and upper corner frequencies, respectively. The digital section is composed of a DSP and a WiFi module. Through its on-board A/D converter, the DSP converts the amplified signals into 12-bit information at a sample rate of 3200 Sa/s; thanks to the oversampling technique, 14-bit data are obtained at a sample rate of 200 Sa/s, corresponding to a resolution of 50 nV. After acquisition, data are sent via UART to the WiFi module, which transmits them to a laptop. The headband is battery-operated with a single LiPo cell and has a capacity of 850 mAh, allowing for ten hours of continuous transmission.

**Figure 2:**
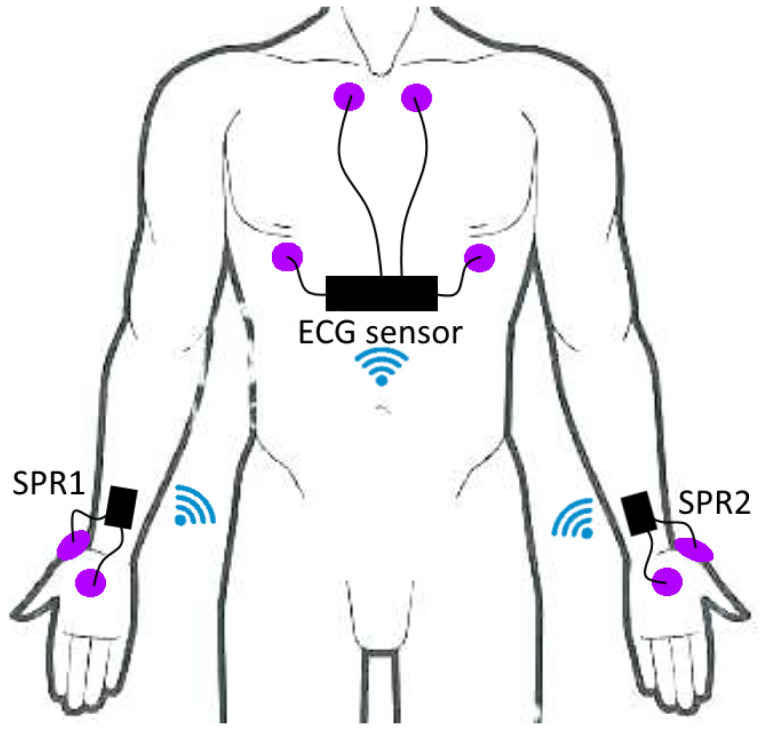
Placement of the ECG and SPR electrodes.

In the second experiment, ECG and EDA signals were acquired in addition to EEG signals; the ECG and EDA sensor systems were developed by the authors and are described in detail in [34]. The system is composed of a dual channel ECG sensor and two EDA sensors. The EDA sensors acquire the SPR by posing two Ag/AgCl electrodes on the palm and back of each hand. We decided to use two sensors, one on each hand, in order to avoid motion artifacts that can arise due to hand motions during driving [34]. Each SPR sensor acquires the low-level differential voltage on each hand (in the ±10 mV range) and converts it into a high-level signal which spans up to 3.3 $V_{pp}$. Then, the information is digitized with a 12-bit resolution and sent to a WiFi module for wireless transmission. The nonlinearity of SPR readings results (as reported in [34]) in their being lower than 30 μV, with a resolution of 5 μV and bandwidth in the [0.08, 8] Hz range. The ECG sensor acquires the low-level derivations on the chest by means of four Ag/AgCl electrodes and converts the low-level differential voltages (in the ±5 μmV range) into 3.3 $V_{pp}$. Afterwards, the data are digitized with 12-bit resolution and sent to a WiFi module. The nonlinearity of the ECG sensor means that the results are on the order of 5 μV, the resolution is 2.4 μV, and the bandwidth is in the [0.08, 75] Hz range. The locations of the electrodes for the ECG and SPR sensors are schematized in Figure 2.

Particular care was taken with the data transmission protocol. It is mandatory to maintain all of the sensors in time alignment and with good accuracy. For this reason, we chose to set the ECG sensor as the access point while the others (EEG, SPR) transmit the data to the access point via an UDP protocol. In this setup, the ECG sensor is responsible for maintaining time alignment between the SPR and EEG data and the ECG data, as well as for transmitting all of the data to a laptop. This choice leads to a maximum time misalignment between signals on the order of just 50 ms [34].

### 2.2. Signal Processing

After acquisition, data were postprocessed in order to extract information on driver attention.

EEG signals were processed with EEGlab, a toolbox for Matlab [35], in order to remove artifacts due to head motion. Subsequently, the spectral components of the EEG signals were divided into the standard bands, namely, delta [0.5, 4] Hz, theta [4, 8] Hz, alpha [8, 12] Hz and beta [12, 30] Hz. The power of each band was computed by integrating the power spectral density over the relevant frequency range. The estimation of power bands was performed by applying the Welch Periodogram on 4 s blocks (i.e., 800 samples) with overlap of 50% (i.e., 400 samples) using the Hanning window. In this work, we concentrated on the power of the beta waves, as in the literature the power in this frequency band is associated with discomfort, stress, and attention [26,36].

Because EEG signals usually present additional artifacts due to other physiological factors such as cardiac and muscle activities, and may be subject to power line interference, we used Independent Component Analysis (ICA) along with the labelling procedure provided by EEGLab. This analysis allows for quantification of the ECG, muscle activity, and power line components within the EEG signals and provides the percentage amount of these artifacts. As reported in detail in Section 4, the overall impact of these artifacts is ultimately not relevant.

**Figure 3:**
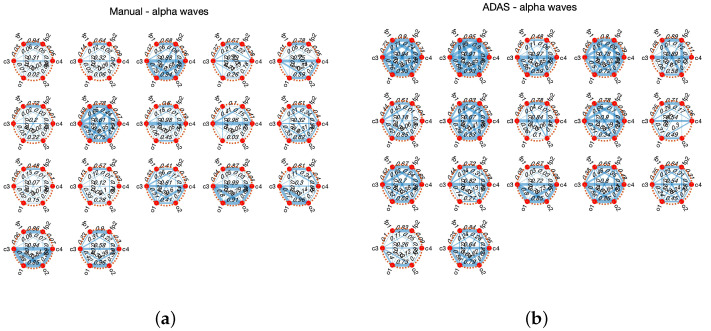
Connectivity networks of subjects 1–17. The thicker lines are associated with higher correlation. (**a**) Manual, alfa waves; (**b**) ADAS, alpha waves.

**Figure 4:**
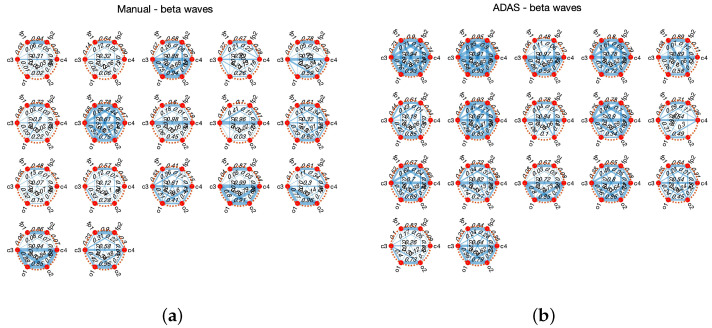
Connectivity networks of subjects 1–17. The thicker lines are associated with higher correlation. (**a**) Manual, beta waves; (**b**) ADAS, beta waves.

For illustration purposes, Figure 3 and Figure 4 show the connectivity networks of the six EEG derivations (Fp1, Fp2, C3, C4, O1, and O2) taking into account the signals acquired from the seventeen subjects involved in the second experiment. Here, we only consider the alpha [8, 12] Hz and beta [12, 30] Hz waves [37], as it is well known (see [26,36]) that the power computed in these frequency bands is related to various mental states such as relaxation, discomfort, and stress. The mutual correlation between EEG channels is used to derive each connectivity matrix. Observing these figures, in particular those with alpha waves, it can be seen that the overall connectivity between channels is lower in the manual driving scenario, indicating that the relevant brain activity could be happening in particular brain regions. With beta waves, the differences in connectivity are less marked.

**Figure 5:**
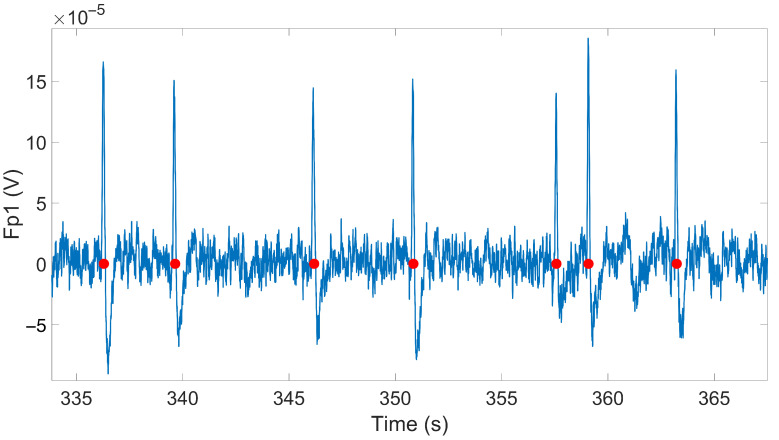
Example of EEG signal in Fp1 location (blue line) and blink identification by Blinker (red markers).

The blink rate was extracted using Blinker, a Matlab toolbox [38] which extracts eye blink locations and statistics (duration, slew rate, EBR) starting from the frontal derivations (Fp1, Fp2) of EEG signals. The extraction of blink statistics is performed by the algorithm, which executes the following steps: (1) FIR band-pass filtering of the EEG signal in the frequency range [1, 20] Hz; (2) marking the portions of the EEG signal where the amplitude is higher than the mean of the signal by 1.5 standard deviations; (3) identifying the portions of the EEG signal (among the ones marked in the previous step) with durations longer than 50 ms and with a minimum distance of 50 ms with respect to the previous and next marked intervals; (4) calculating the blink parameters (position, peak, rise time, fall time, slew rate); (5) calculating the correlation of the identified blinks to a stereotypical blink; (6) eliminating the identified blinks with low signal-to-noise ratios; and (7) eliminating other eye movements from the identified blinks. Figure 5 shows an example of blink position extraction (red markers) from the Fp1 channel EEG signal (blue line). Subsequently, the EBR is obtained as the total number of detected blinks divided by the total experiment duration.

The ECG signal is processed in order to extract the heart rate (HR) and heart rate variability (HRV). In particular, the ECG signal is processed with the Pan-Tompkins algorithm in order to find the R-peaks in the QRS complex. The time between two subsequent peaks is the instantaneous RR interval (which is resampled at 200 Hz), and its inverse represents the instantaneous HR. In this work, it should be noted that when we manually checked the ECG signals, no ectopic beats appeared; otherwise, we would have had to eliminate the ectopic beats and replace the instantaneous ectopic HR with the average between the previous and the next values having normal sinus rhythm.

Regarding the SPR, we evaluate the signals coming from two sensors (SPR1 and SPR2 in Figure 2). These are processed via the motion artifact removal algorithm we previously presented in [39]. The motion artifact removal algorithm assumes that the SPR pulses, which are correlated with autonomic nervous system activity, are similar in both cases, while the spurious signals caused by hand motions generate an asymmetric energy increase mainly visible in one hand at a time (i.e., the hand which is mostly engaged in the physical action). Thus, the algorithm computes the energy of the two SPR signals over a moving window with a duration of 1 s, and tends to follow the input signal with lower energy if there is discordance between signals. The output of the algorithm then results in a single SPR signal without motion artifacts. Finally, the root mean square (RMS) value of the output signal is computed, and this quantity is used to assess the electrodermal activity.

### 2.3. Statistical Tests and Methods

The Gaussianity of data samples is a typical assumption in situations where accurate information on data statistics is lacking, as is typically the case for small data samples. We performed Gaussianity tests on our data using the Kolmogorov–Smirnov test, assuming, though it is not wholly correct, that the mean and variance of the data were equal to those of the samples. In addition, we performed the more accurate Lilliefors test, in which critical values are calculated using Monte Carlo simulations and assuming unknown mean and variance. All the acquired data passed the Kolmogorov–Smirnov Gaussianity test at the α=0.05 significance level, while all the data except the beta power in the Manual scenario passed the Lilliefors test for Gaussianity (α=0.05).

To quantify the hypothesis that data characteristics change in the different scenarios, in Section 3 we report the results of both the *t*-test, which assumes Gaussianity, and of the more general non-parametric Wilcoxon test. The paired *t*-test is a parametric test used when the same subject performed a task two times, and was used to verify whether there was a significant difference between data characteristics in the first and second task.

The Wilcoxon test relies on the sum of the rankings of the input data to verify whether the data distributions in the first and second task are significantly different. In contrast to the *t*-test, the non-parametric Wilcoxon test does not assume Gaussianity.

Both tests provide as output a probability *p* that the two sets of data belong to different distributions. As commonly accepted in the literature, we assume a good level of significance if the probability satisfies p≤0.05. These values are highlighted in bold in the tables in Section 3. Such a result means that when p≤0.05, it can reasonably be assumed that there is a significant difference between the values in the first test setup as compared to the second test setup. For the first experiment, we performed the Kruskal–Wallis test to compare the three groups, confirming the hypothesis that the samples do not have the same distribution. We believe that comparing the samples in pairs can provide even clearer evidence of the differences among data characteristics.

## 3. Experimental Setup

In this work, we analyze physiological data acquired from healthy subjects during two different experiments carried out in our BioSensLab laboratory at the University of Udine [40]. The participants were provided with a guideline document asking them to not drink coffee or other beverages with caffeine and to not smoke for at least two hours before the experiment. They had to be in possession of a driver’s license. In addition, none of the subjects had any history of psychiatric or neurological illnesses, and all had normal or corrected vision.

**Figure 6:**
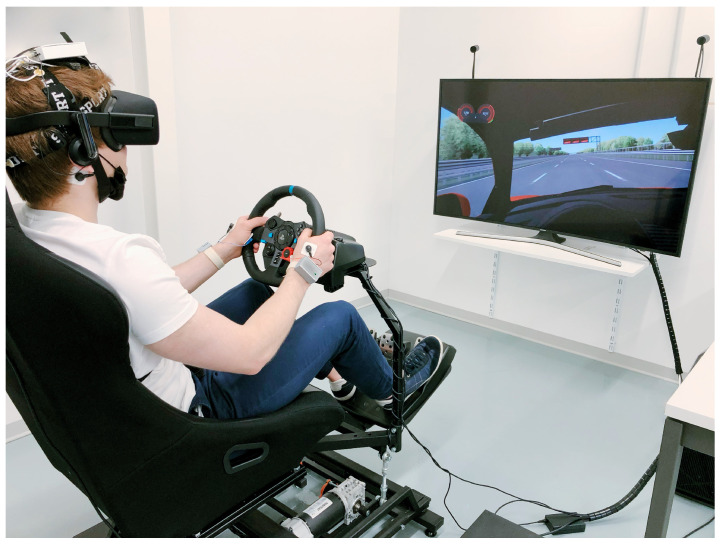
A test subject in our lab using our simulator setup, with SPR, ECG, and EEG sensors employed to acquire the physiological signals (Experiment 2).

In the first experiment, we collected EEG signals from the subjects and derived the blink rate from this in order to first evaluate whether and how this signal alone could reveal possible changes in the mental attention state of the subjects when comparing the manual and autonomous scenarios. In the second experiment, we recorded SPR, ECG, and EEG signals from a different group of subjects. In this case, we report the results obtained by using these three signals together in a multisensor acquisition setup. The driving simulator used in both experiments consisted of a three-axis moving platform (DOF Reality Professional P3) controlled by driving software, allowing the subjects to feel the car movements in both manual and autonomous driving modes. The platform, along with a curved screen, Virtual Reality (VR) (Oculus Rift) headset, force-feedback steering wheel, pedals, gearbox (Logitech G29), and racing seat (see Figure 6), were connected to a PC running the driving software. The subjects wore the VR headset throughout the tests to ensure that they were not influenced by glare or lighting conditions in the environment. Each subject performed all of the test phases one after the other in a single session. Both experiments were carried out according to the principles of the Declaration of Helsinki, as described them in detail below.

**Figure 7:**
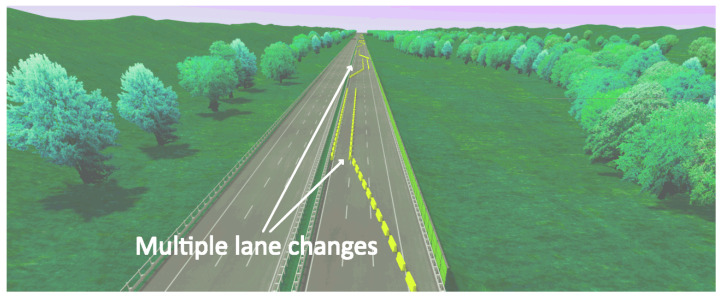
Example of obstacle mimicking road work with Jersey barriers to force multiple lane changes.

Experiment 1: Ten subjects took part to this experiment, seven men and three women. Their age was in the (29 ± 5) range, with average driving experience of 11 years. They were asked to use the simulator and drive along a highway with Jersey barriers placed along it in well-defined locations. More specifically, the course was 20 km long with 10 Jersey barriers placed at 2 km distances one from the other, the first being at 2 km from the beginning of the track. These barriers were meant to simulate road work, and involved lane changes or road narrowing which the subjects had to overcome (see Figure 7). 

The subjects had to complete the track three times (i.e., in three separate sessions), each time in a different driving scenario: a manual driving scenario (denoted as “Manual” from now on), an autonomous driving scenario with a cautious algorithm set on the driving simulator (denoted as “ADAS1”), and finally an autonomous driving scenario with an erratic algorithm set on the driving simulator (denoted as “ADAS2”). In ADAS1, the frontal acceleration of the vehicle while driving was set to 8 m/s^2^ and the lateral acceleration was set to 3 m/s^2^. In ADAS2, the vehicle acceleration was not specified except for the limits of the vehicle dynamics. For each subject, the order of these three sessions was randomly chosen. Because we asked that the average velocity kept by each subject be 120 km/h, each simulation lasted 7 to 10 min. In addition, in the Manual scenario the subjects were encouraged to drive responsibly, as in a real-world scenario, while trying to avoid speeding and accidents. In the autonomous scenarios the subjects only needed to experience the drive and the platform movement, looking at the road when they wanted while the software (with either cautious or erratic behaviour) drove the car autonomously along the track. During this first experiment only EEG data were acquired from the EEG headband and transmitted to a computer via WiFi using TCP protocol. We developed a Graphical User Interface (GUI) that allowed us to gather, display, and save the data before further processing.

Experiment 2: Seventeen subjects (15 men and 2 women) participated in the experiment, in the 20–37 age range, with 7 years of average driving experience. They signed informed consent that allowed us to acquire their SPR signals from both hands, their ECG from the chest, and their EEG signals from the head throughout the experiment. At first, they were asked to sit in a chair in front of a PC monitor (not the simulator) and look at it while it displayed a single black point at the center. This phase was meant to relax and calm the subjects; at the same time, it allowed us to record signals that might be useful as a baseline, in particular for comparison of the same physiological signals among different subjects. Then, similarly to the previous experiment, the participants were instructed to drive along a straight 14 km highway in the simulator in a full manual driving scenario (referred to as “Manual” from now on). During this phase, they had to overcome six tasks while driving. In particular, six portions of the road had road work on it, defined by Jersey barriers which were added beforehand. Each task spanned 200 m, and the distance between them was 2 km. The first was located at 2 km from the start of the track, and there were 800 m left in the track after the last task. At an average velocity of about 120 km/h, this phase lasted about 7 min. Finally, the participants had to experience a drive along the same highway, again with six tasks, now in an autonomous driving scenario (referred to as “ADAS”). These six tasks were chosen to be different from the ones selected for the manual phase. During this experiment, SPR, ECG, and EEG sensors were used to log data, all of which were sent to a computer through WiFi, as in the previous experiment; the only difference in data transmission with respect to previous experiment was that here we used ECG sensor as an access point and the data were transmitted (via UDP protocol) from the EEG and SPR sensors to the ECG sensor, which then transmitted all the data to a laptop. We developed a new GUI which enabled us to collect all signals synchronously, monitor them in real time, and save them.

## 4. Experimental Results

In this section, we present the results obtained by processing the data logged from the subjects during the two experiments described above. An in-depth discussion of these results is provided in Section 5.

**Figure 8:**
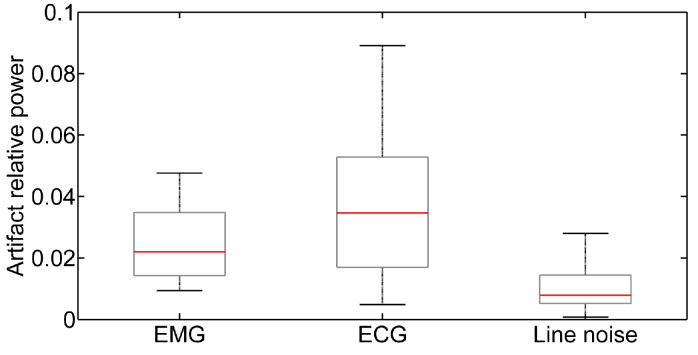
Box plot representing the relative power content of muscle (EMG), heart (ECG), and power line noise for all of the recorded signals.

As introduced in Section 2.2, in order to quantify the impact of possible artifacts on the EEG signal we performed Independent Component Analysis (ICA) on the EEG signals using EEGLab. Figure 8 shows a box plot of the relative power content relevant to muscle activity, ECG, and power line noise for all of the signals acquired from the subjects.

It can be seen that the power line provides the lowest contribution (on the order of 0.8%) thanks to the digital notch filter implemented on the GUI, which has 30 dB power line suppression, while muscle activity (EMG) and ECG manifest a slightly higher relative power, on the order of 2% and 3.5%, respectively. In Figure 8, the lines dividing the boxes into two parts represent the medians, the boxes represent the 25th and 75th percentiles, and the whiskers are based on computation of the interquartile range. As is apparent, the impact of the artifacts is small.

The data were then processed for each experiment. For Experiment 1, we only take into account the EEG signals logged from each subject, analyzing the detected blinks with the related EBR (i.e., the ratio between the number of blinks detected by Blinker and the time needed by the subject to complete the course in minutes), along with the power of the EEG beta waves. The average of these parameters is compared among subjects during each driving scenario (Manual, Autonomous 1, and Autonomous 2). Further statistical tests were carried out to compare the EBR and the EEG beta power of the ten subjects. In Experiment 2, we focus on the analysis of the EBR, EEG beta power, RMS of the SPR signal, and mean of the HR signal. More specifically, these values are computed considering the entire signals logged from the the whole track, for each subject. After that, we calculate the average of these values considering all of the test subjects and compare the results among the various driving scenarios, i.e., Manual and ADAS. Statistical tests were applied in this case as well, this time considering the derived parameters for the seventeen subjects in the two scenarios.

### 4.1. Experiment 1

Here, we summarize the results of the first experiment, which was partially described in [27]. Table 1 shows the EBR and average beta power of the O2 channel for all scenarios and each subject.

This table shows that EBR varies quite noticeably from subject to subject. In addition, it shows quite well that EBR is reduced during manual driving compared to the two scenarios with autonomous driving, reflecting the increased attention associated with manual driving. Table 1 reports the power of the EEG beta waves, which correspond to the EEG spectral content in the range of [12, 30] Hz. As mentioned above, the beta power is evaluated integrating (over the frequency range of [12, 30] Hz) the Welch-periodogram power spectral density estimate of the EEG signal of the O2 derivation. The estimate is computed by dividing the signal into blocks of 4 s duration windowed with the Hanning window and with 50% overlap. It can be noted that the beta power is higher during manual driving as opposed to the two autonomous driving scenarios.

**Figure 9:**
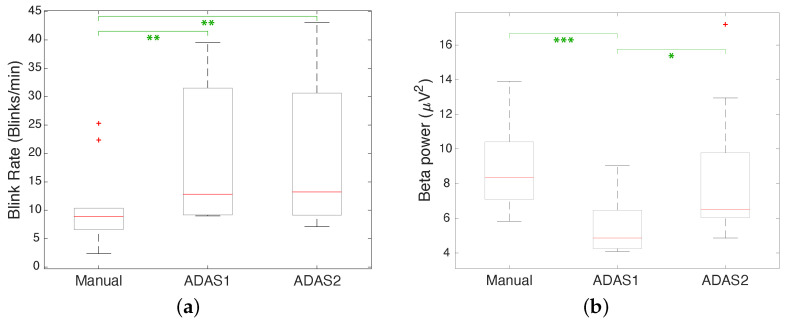
(**a**) Box plot of EBR in Manual, ADAS1, and ADAS2 scenarios; (**b**) box plot of beta power in Manual, ADAS1, and ADAS2 scenarios. The red lines correspond to the medians, the boxes correspond to the first and third quartiles, the whiskers correspond to the values based on the Interquartile Range (IQR) computation, and the red plus sign markers correspond to the outliers. The green asterisks refer to the *p*-values computed using *t*-tests.

For a better comparison, Figure 9 shows the box plot of EBR and beta power, taking into account the data of all the subjects in the three scenarios. The box plot in Figure 9a shows that EBR is considerably lower during manual driving than during the ADAS1 and ADAS2 scenarios. In addition, the box plot in Figure 9b shows that the beta power is higher during manual driving compared to the ADAS1 and ADAS2 scenarios. For each subject, we computed the ratio between the EBR in the manual scenario and the corresponding average of the values in the two autonomous scenarios and did the same for the beta power. For the EBR, the mean of the values of this ratio was 0.57, with a standard deviation of 0.21, while for the beta power the mean was 1.33 with a standard deviation of 0.61. These values again confirm that the drivers were more engaged during manual driving, with a reduced EBR and increased beta power.

A quantitative evaluation of the previous observations can be obtained by carrying out two statistical tests, i.e., a parametric test (*t*-test) and a non-parametric test (Wilcoxon signed rank test). Table 2 shows the results of these tests in terms of the *p*-value when comparing the EBR and beta power values in the ADAS1 vs. ADAS2, Manual vs. ADAS1, and Manual vs. ADAS2 scenarios. When considering EBR, both the *t*-test and Wilcoxon test show that the EBR in ADAS1 appears to be higher in a significant way than in Manual, while the EBR in ADAS2 appears to be higher in a significant way than in Manual and the EBR in ADAS1 and ADAS2 provides similar results. When considering the EEG beta power, it can be seen that the *t*-test and Wilcoxon test point to the conclusion that ADAS1 is less engaging in a significant way than ADAS2, ADAS1 is less engaging in a significant way than Manual, and ADAS2 and Manual produce similar results.

The box plots in Figure 9 are annotated with asterisks according to the *p*-values computed using the *t*-test. We followed the common convention in denoting *p*-values with * when p≤0.05, with ** when p≤0.01, with *** when p≤0.001, and with **** when p≤0.0001.

### 4.2. Experiment 2

In Table 3, we show the EBR, beta power, SPR RMS, and mean HR computed for each subject and each scenario in the second experiment. In addition, in the final row we show the mean of these parameters as obtained by averaging the values corresponding to all of the subjects.

The parameters can differ significantly from a subject to another. However, when looking at the mean EBR, it can be noted as a result of considering all subjects that it is lower in the manual driving scenario than in the autonomous scenario.

**Figure 10:**
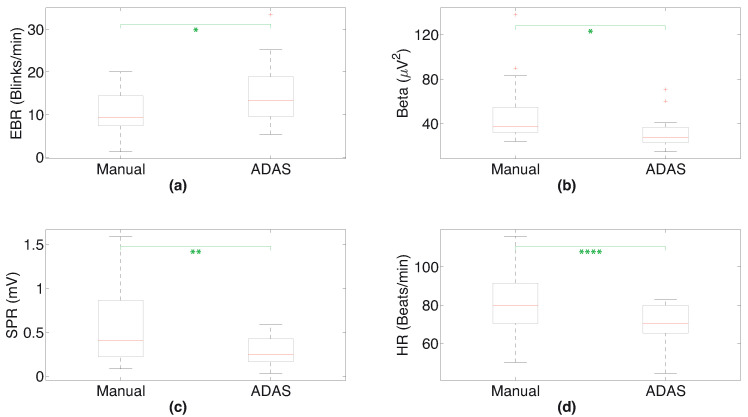
(**a**) Box plot of EBR among subjects in Manual and ADAS scenarios; (**b**) box plot of beta power; (**c**) box plot of SPR RMS; (**d**) box plot of HR. The green asterisks refer to the *p*-values computed using *t*-tests.

**Figure 11:**
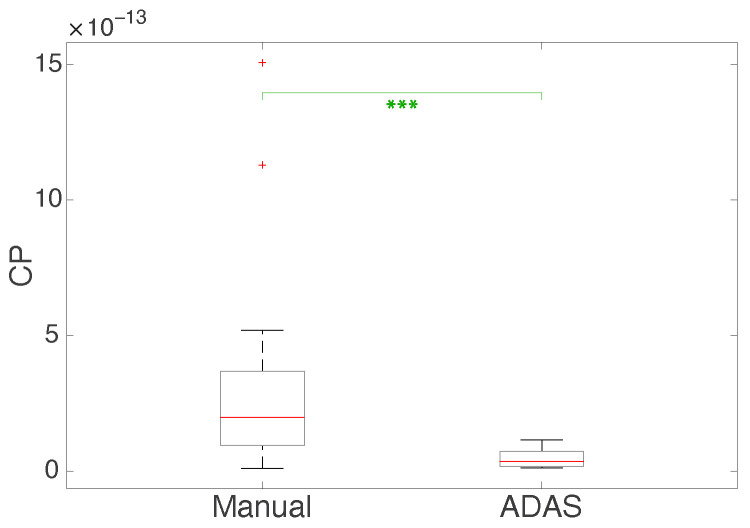
Box plot of the CP parameter (SPR RMS × mean HR × beta power/EBR, with SPR in mV, HR in Beats Per Minute, beta power in μV^2^, and EBR in Blinks Per Minute) among subjects in the Manual and ADAS scenarios. The red lines correspond to the medians, the boxes to the first and third quartiles, the whiskers to the values based on the Interquartile Range (IQR) computation, and the plus sign markers to the outliers. The green asterisks refer to the *p*-values computed with the Wilcoxon test.

This result is revealed from the box plot reported in Figure 10a as well, showing how the EBR in the manual setting is lower and much different from the autonomous setting. By analyzing the beta power of the EEG waves reported in Table 3 (and in the box plot in Figure 10b), it can be observed that the mean beta power is higher in the manual scenario than in the ADAS scenario. In the end, looking at the mean of the RMS of the SPR signals and the mean of the HR computed by averaging the values of all of the test subjects, it is evident that these measurements are higher in the manual scenario than in ADAS (see Figure 10c,d). In Figure 11, we show the box plot of the values calculated by multiplying the SPR RMS, HR mean, and beta power values (which all increase from Manual to ADAS) and dividing by EBR (which decreases from Manual to ADAS) for each subject in the two different scenarios; we indicate this parameter as “CP”.

As in the previous experiment, we carried out an additional analysis considering the *t*-test and the Wilcoxon test. More specifically, we compared the values of the seventeen subjects’ SPR RMS, mean HR, EEG, and beta power parameters for each scenario. The results are presented in Table 4 and Table 5 for the *t*-test and Wilcoxon test, respectively. From both tables, it can be seen that in the majority of cases there is a significant difference between the parameters calculated when considering the different driving scenarios in both tests. Regarding the Wilcoxon test, Table 5 includes the results obtained considering the CP parameter of all subjects in the two different scenarios. It can be observed from Table 5 that there are two cases in which the probability *p* is greater than 0.05, which happen when comparing the EBR and SPR RMS among the subjects (although the probability *p* related to the SPR RMS measurement is slightly greater than the 0.05 threshold). The box plots in Figure 10 and Figure 11 include asterisks according to the *p*-values computed using the *t*-test and the Wilcoxon test, respectively. We follow the same convention as before, denoting *p*-values with * when p≤0.05, ** when p≤0.01, *** when p≤0.001, and **** when p≤0.0001.

## 5. Discussion

In this section, we discuss the results introduced in the previous section. In addition, we highlight the key aspects and main advantages of our system, then report limitations and possible drawbacks.

### 5.1. Experiment 1

First, we consider the first experiment. Table 1, reporting the EBR and beta power values for all of the test subjects, shows that EBR can change significantly from individual to individual. The EBR can in fact be affected by several biological factors, for example cognitive or visual capacity, as well as personal traits such as age, health, and lifestyle factors [41,42]. In addition, the EBR can change during the day, i.e., at certain hours of the day it can be different than at other hours [43]. From this table, it can be noted that the EBR is lower in the manual driving scenarios as opposed to the autonomous driving scenarios, highlighting the higher attention level of the subjects while driving manually. This finding is in agreement with what is reported in the literature, for example in [44], where the value of EBR decreases when performing dynamic visual assignments. Table 1 shows the EEG beta power. Again, this is more prominent when driving in the manual scenario and manifests a lower value during the two autonomous driving scenarios. As noted before, this is in accordance with previous papers presented in the literature which have shown that the spectral components of the EEG signal become greater in individuals experiencing high mental concentration or going through distracting events compared to other less mentally engaging situations. These results are further confirmed by the box plot of EBR and beta power displayed in Figure 9. Qualitatively, this figure shows a noticeable difference between the values observed in the manual driving scenario compared to the autonomous driving ones, especially as regards the EBR. In particular, it can be noted that the blocks that delimit the 25–75 percentiles do not overlap. As for the beta power, there is a clear difference between Manual and ADAS1, though this difference is less evident when considering ADAS2, which corresponds to more aggressive autonomous driving. The results of the *t*-test and the Wilcoxon signed rank test are reported in Table 2, evaluating the EBR and beta power values in ADAS1 vs. ADAS2, Manual vs. ADAS1, and Manual vs. ADAS2 scenarios. As introduced before, when evaluating EBR both tests indicate that the EBR in ADAS1 is significantly higher than in Manual, that the EBR in ADAS2 is significantly higher than in Manual, and that the EBR values in ADAS1 and ADAS2 provide comparable results. When evaluating the EEG beta power, on the other hand, both tests indicate that ADAS1 is significantly less engaging than ADAS2, that ADAS1 is significantly less engaging than Manual, and that ADAS2 and Manual provide comparable results. The results of this experiment, along with those presented below for the second experiment, confirm that the measurements obtained through analysis of the EEG signal, in particular when considering the blink rate and the beta power, provide consistent results and can be used as a good indicator of driver attention levels. Because the use of the EEG signal alone is sufficient for blink rate detection, cameras are not necessary, avoiding any problems with posture and lighting. Furthermore, analysis of EEG signals allows us to obtain useful additional information, such as the beta power considered in this paper.

### 5.2. Experiment 2

As far as the second experiment is concerned, Table 3 reports the EBR, the beta power, the SPR RMS, and the mean HR computed for each subject and each scenario, including the means of these parameters. Again, it can be noted that the parameters vary appreciably among individuals. In particular, as already mentioned, the EBR may depend on various aspects related to visual functionality as well as to the age and gender of the subject under test. Nonetheless, the mean of the EBR is smaller in the manual driving scenario than in the autonomous scenario, suggesting that when driving manually the subjects experienced a greater mental load and needed to concentrate more on overcoming the obstacles, resulting in lower blink activity. This is in line with the results obtained in the first experiment. From Table 3, it can be seen that the mean beta power is again bigger in the manual scenario than in the ADAS. These results are confirmed by the box plot in Figure 10a, where the EBR in the manual scenario appears smaller and has a different value compared to the autonomous scenario. The RMS of the SPR signals and the mean of the HR signals are larger in the manual scenario than in the ADAS scenario, as shown in Figure 10c,d. In our previous papers [45,46], we have demonstrated that both SPR and HR signals typically increase when individuals undergo stress episodes or are engaged mentally, and are higher when evaluated on a whole track with obstacles placed on it during a manual drive than on an autonomous one. To sum up, we found that the SPR RMS, mean HR, and beta power values increase in this experiment during the manual driving, whereas the EBR is the only parameter that decreases in the manual scenario. For this reason, in Figure 11 we show the box plot of the values obtained by computing the product of the SPR RMS, HR mean, and beta power values, as they are higher in Manual than in ADAS, while dividing by the EBR, as it is lower in Manual than in ADAS. The separation of the values between the two scenarios is even more remarkable. Table 4 and Table 5 show the results of the *t*-test and the Wilcoxon test of this experiment, evaluating the statistical significance among the data belonging to all of the subjects in the manual and autonomous scenarios. In detail, the *t*-test shows that the SPR RMS, mean HR, and beta power parameters are significantly higher in the Manual scenario than in the ADAS scenario, while the EBR is significantly higher in the ADAS scenario than in the Manual scenario. This is in accordance with the box plot representations displayed in Figure 10, because boxes which are clearly in different positions (without overlaps) are an indication of smaller *p*-values, whereas boxes with large overlaps are an indication of higher *p*-values. Regarding the Wilcoxon test, Table 5 includes the results obtained for the CP parameter. We cannot apply the *t*-test using these parameter values, as the *t*-test assumes that data are occurrences of independent normal random variables, and this hypothesis is no longer true when we consider values from a composition of multiple operations (such as products and divisions). As already stated, there are two cases in Table 5 with a probability *p* greater than 0.05, appearing when we compare the EBR and the SPR RMS among the subjects (the probability *p* related to the SPR RMS measurement is, however, slightly higher than 0.05). To sum up, we can state that the physiological measurements computed taking into consideration all of the subjects are significantly different when evaluating them in the two driving scenarios, suggesting that these scenarios actually lead to different attention levels in the subjects under test.

### 5.3. Strengths and Weaknesses of Our System

Most of the systems proposed in the literature are based on analysis of images and video sequences, and have the aim of identifying certain visual elements such as the blink rate, face or head direction, or other particular behaviours of the driver. For instance, in [47] the authors introduced a system with a camera to track the head and face of the driver using image processing techniques. The proposed system was able to capture the driver’s eye blink and head rotation to evaluate their attention level. Similarly, in [48] the authors presented a visual monitoring system to evaluate the fatigue and monotony state of drivers. The system used a low cost camera to detect head direction and blink patterns. Another study [49] employed a GPS and a two-axis accelerometer integrated with three cameras to monitor the driver and their surroundings, with the main objective of evaluating driver performance.

These systems are prone to problems related to possible light variations, non-ideal posture of the subjects, and interference in the scene from objects of various kinds such as glasses or accessories. On the contrary, the possibility of acquiring physiological signals correlated to the psycho-physical state of the driver, as considered in the present work, could allow for more consistent assessment. Moreover, the use of a camera may not be applicable in certain situations due to privacy and ethical reasons or high costs. Wearable bio-sensors are prone to movement artifacts, and similar to camera-based solutions can be influenced by vibrations and movements. In addition, as already mentioned, individual and subjective matters (e.g., sudden changes in light that may affect blink rate, or other causes that affect HR and SPR) can alter the acquired physiological signals for reasons beyond driver attention or events related to driving. Therefore, it is not easy to unambiguously define possible alert thresholds for drivers. Nonetheless, the system can certainly provide useful information on the psycho-physical state of drivers in order to suggest possible countermeasures. The wearability of our proposed system has the advantage of higher accuracy, along with the disadvantage of being slightly intrusive for the driver, while camera-based solutions are contactless and independent from the driver. To solve the problem of intrusiveness, non-wearable biological sensors have been presented in the literature, for instance, sensors integrated in the driver’s seat such as capacitive electrocardiogram (cECG), Ballistocardiogram (BCG) [50,51], Seismocardiogram (SCG) [52], or steering wheel ECG [53], or radar systems [54]. However, compared to wearable bio-sensors they have less efficiency and accuracy and are more prone to being affected by other environmental conditions such as humidity, vibration, and the driver’s clothing [55].

Clearly, each system has its advantages and disadvantages. The choice of the most appropriate system depends on the requirements of the application, environmental conditions, and available budget. In general, a sensor fusion approach combining camera-based and physiological measurements could be a good solution in the context of advanced driver monitoring systems, supporting and improving the results obtained by each system. Our sensors are currently designed as prototype laboratory tools for further tests using driving simulators. When considering possible practical implementation of the scheme for use in the real word, there are potentially simpler solutions for biological signal acquisition, for example, by acquiring EEG through glasses or hats, EDA from gloves, and ECG from the seat.

## 6. Conclusions

In this paper, we have proposed a scheme for monitoring driver attention based on the acquisition of their EEG, SPR, and HR signals. In particular, estimation of the eye blink rate was performed automatically by analyzing EEG signals. Our experiments were organized in two phases. In the first experiment, we considered only the EEG signal, comparing it in a manual driving and two different autonomous driving sessions. We observed that the EBR was lower during manual driving compared to autonomous driving, confirming the increased attention of the subjects while driving manually. The power of the EEG beta waves appeared to be higher during the manual scenario as well, which again was associated with higher mental engagement on the part of the drivers. In the second experiment, the EBR, EEG beta power, RMS of the SPR signal, and mean of the HR signal were considered during two driving sessions, one manual and one autonomous. The same considerations as in the first experiment on only the EBR and the EEG beta power features were confirmed in the second experiment as well, which additionally revealed that the RMS of the SPR signals and the mean of the HR signals were generally higher in the manual scenario than in the autonomous one. In both experiments, therefore, the proposed system was able to discriminate the different driving conditions by associating a state of increased attention with manual driving. The significance of the data was confirmed through the use of appropriate statistical tests. In summary, the proposed system based solely on the acquisition of biophysical signals seems to offer good prospects for the recognition of different states of attention, and more generally of the psycho-physical state of the driver.

## Figures and Tables

**Table 1:** Blink rate (FP1 derivation) and beta power (O2 derivation) for all scenarios and each subject.

	Manual		ADAS1		ADAS2	
Subject	EBR ${}^{a}$	Beta Power ${}^{b}$	EBR	Beta Power	EBR	Beta Power
1	2.36	8.29	9.01	4.68	9.13	6.63
2	25.31	8.71	31.53	6.90	43.08	9.77
3	8.82	13.89	12.14	5.05	9.13	4.86
4	22.37	6.07	39.53	4.25	41.58	5.55
5	10.37	10.41	9.90	6.46	15.99	6.31
6	7.03	7.09	9.20	4.63	9.92	7.10
7	9.95	8.38	34.37	5.24	30.65	6.05
8	5.74	7.10	9.17	4.10	7.13	17.19
9	5.74	7.10	21.03	9.03	15.03	12.94
10	6.60	5.83	13.51	4.08	11.47	6.37

^a^ Blinks Per Minute. ^b^ μV^2^.

**Table 2:** Wilcoxon test and paired *t*-test probabilities.

EBR *p*-Value			
	**ADAS1 vs. ADAS2**	**Manual vs. ADAS1**	**Manual vs. ADAS2**
*t*-Test	0.83	**0.008**	**0.006**
Wilcoxon	0.969	**0.038**	0.054
**Beta Power *p*-Value**			
*t*-Test	**0.044**	**0.001**	0.797
Wilcoxon	**0.025**	**0.003**	0.326

Values in bold highlight a good level of significance (*p* ≤ 0.05).

**Table 3:** EBR, mean beta power, SPR RMS, and mean HR computed for each subject and each driving scenario.

Subject	Manual				ADAS			
	EBR ${}^{a}$	Beta Power ${}^{b}$	SPR RMS ${}^{c}$	Mean HR ${}^{d}$	EBR	Beta Power	SPR RMS	Mean HR
1	16.39	37.45	1.05	100.72	25.27	40.47	0.59	82.95
2	6.64	42.07	0.22	79.29	16.52	29.59	0.35	70.31
3	5.93	33.48	0.38	69.55	5.36	14.54	0.10	63.53
4	19.29	33.47	0.08	69.97	18.07	24.49	0.22	59.06
5	20.01	54.38	0.87	70.56	13.41	21.68	0.42	69.41
6	11.99	26.13	0.16	88.51	17.22	25.55	0.29	76.57
7	9.23	29.75	0.18	77.17	5.65	30.31	0.03	69.04
8	10.10	89.73	0.27	76.50	10.84	60.21	0.24	70.16
9	4.02	23.70	0.86	50.14	9.60	20.73	0.12	44.30
10	7.55	32.35	1.02	84.91	9.46	25.79	0.17	77.64
11	1.25	50.85	0.32	115.79	5.36	23.20	0.23	78.54
12	8.07	30.91	0.67	79.52	13.27	27.32	0.51	66.04
13	7.73	52.85	1.59	104.07	21.17	36.77	0.43	80.88
14	19.29	54.54	0.84	83.56	23.30	70.60	0.47	80.19
15	8.47	82.87	0.41	91.40	11.01	31.91	0.15	80.09
16	13.71	137.82	0.79	65.02	33.44	36.71	0.24	58.84
17	12.44	35.06	0.21	91.40	12.85	19.91	0.23	79.73
**mean**	**10.71**	**49.85**	**0.58**	**82.24**	**14.81**	**31.75**	**0.28**	**71.02**

^a^ Blinks Per Minute; ^b^ μV^2^; ^c^ mV; ^d^ Beats Per Minute.

**Table 4:** Paired *t*-test probability *p*-value considering Manual versus ADAS scenarios using different physiological measurements (EBR, beta power, SPR RMS, and mean HR) computed for all subjects.

Measurement	Manual vs. ADAS
EBR	**0.017**
beta power	**0.013**
SPR RMS	**0.004**
mean HR	**0.00006**

**Table 5:** Paired Wilcoxon test probability *p* considering Manual versus ADAS scenarios using different physiological measurements (EBR, beta power, SPR RMS, mean HR, and SPR RMS × mean HR × beta power/EBR) computed for all subjects.

Measurement	Manual vs. ADAS
EBR	0.12
beta power	**0.008**
SPR RMS	0.054
mean HR	**0.033**
SPR RMS × mean HR × beta power/EBR	**0.0004**

## Data Availability

The data presented in this study are available on request from the corresponding author.

## Abbreviations

The following abbreviations are used in this manuscript:

SPR	Skin Potential Response
EDA	Electrodermal Activity
EEG	Electroencephalogram
ECG	Electrocardiogram
HR	Heart Rate
EBR	Eye Blink Rate
RMS	Root Mean Square
GUI	Graphical User Interface
